# Underutilization of information and knowledge in everyday medical practice: Evaluation of a computer-based solution

**DOI:** 10.1186/1472-6947-8-50

**Published:** 2008-11-05

**Authors:** David Zakim, Niko Braun, Peter Fritz, Mark Dominik Alscher

**Affiliations:** 1 IDM Foundation Institute of Digital Medicine, Am Kriegsbergturm 44, D-70192 Stuttgart, Germany; 2 Department of General Internal Medicine and Nephrology, Stuttgart, Germany

## Abstract

**Background:**

The medical history is acknowledged as the *sine qua non *for quality medical care because recognizing problems is pre-requisite for managing them. Medical histories typically are incomplete and inaccurate, however. We show here that computers are a solution to this issue of information gathering about patients. Computers can be programmed to acquire more complete medical histories with greater detail across a range of acute and chronic issues than physician histories.

**Methods:**

Histories were acquired by physicians in the usual way and by a computer program interacting directly with patients. Decision-making of what medical issues were queried by computer were made internally by the software, including determination of the chief complaint. The selection of patients was from admissions to the Robert-Bosch-Hospital, Stuttgart, Germany by convenience sampling. Physician-acquired and computer-acquired histories were compared on a patient-by-patient basis for 45 patients.

**Results:**

The computer histories reported 160 problems not recorded in physician histories or slightly more than 3.5 problems per patient. However, physicians but not the computer reported 13 problems. The data show that computer histories reported problems across a range of organ systems, that the problems detected by computer but not physician histories were both acute and chronic and that the computer histories detected a significant number of issues important for preventing further morbidity.

**Conclusion:**

A combination of physician and computer-acquired histories, in non-emergent situations, with the latter available to the physician at the time he or she sees the patient, is a far superior method for collecting historical data than the physician interview alone.

## Background

Everyday medical practice does not proceed on the basis of up-to-date information about patients nor on up-to-date knowledge for managing problems that are identified [1-4]. History-taking, the acknowledged starting point for quality medical care, is inadequate so that information about patients goes undiscovered [5-9]; and outcomes from treating recognized problems are unequal to what can be achieved through effective use of existing knowledge [1-3,10,11]. This underutilization of information about patients and knowledge about management appears to be a major contributor not only to poor outcomes but to the high cost of health care [10]. Short-comings in delivering quality care are unlikely to resolve until the ineffective use of information about patients and knowledge about disease are addressed directly. Since information about patients is pre-requisite for effective use of knowledge to manage their medical problems, it makes sense to address first the issue of poor history-taking.

History-taking, in theory, can be off-loaded from physicians to computers, i.e., the data fields of an "electronic medical record" can be populated by direct, computer-based interview of patients. Computerized history-taking was an active area of academic inquiry at the beginning of the computer age; and early literature demonstrates that history-taking by computer was a promising and effective technology (see reference [12] for review). Paradoxically, interest in the technology ebbed as computers became cheaper and easier to use and software easier to write. We believe computing can resolve the negative impact on quality of care from deficiencies in history-taking and that this technology thus should be pursued vigorously. We report experience here with a computerized history-taking program that interacts directly with patients in the absence of inputs from physicians or other providers. The data document the extent to which medical problems are not recorded in routine medical histories and show that computerized history-taking can resolve deficiencies in acquiring information from patients about significant medical problems affecting them.

## Methods

### Software program

**Figure 1:**
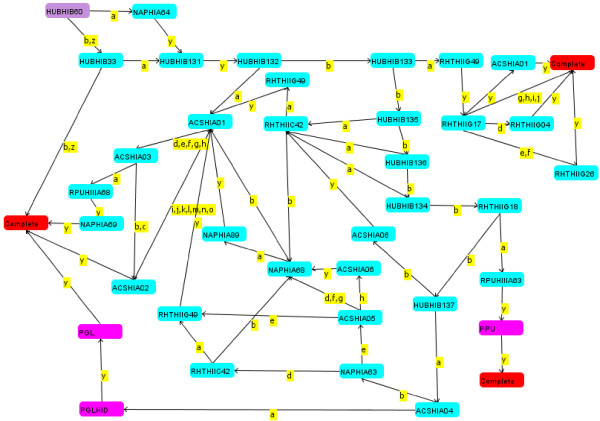
**Example of a decision tree that organizes the flow of questions during an interview**. See text in Methods for a detailed explanation of the tree.

Acquisition of clinical data in the history-taking program is based on the principles of pathophysiology formalized as software algorithms representing medical knowledge as branched chain decision trees. Figure 1 is an example of a typical decision tree, which is the initial tree in the evaluation of a patient presenting with chest pain. The tree in Figure 1 is one of a family of 29 trees used to evaluate patients with chest pain and then to acquire a complete cardiovascular history. The arrangement of nodes in Figure 1 is based on a logical flow of questions in response to answers concerning the pathophysiology of coronary artery disease as it applies to acquiring a history of a patient with suspected coronary disease, as for example the severity, nature, location and radiation of pain, associated cardiac and/or vagal symptoms, precipitators of pain and so on. Nodes coded with 4 letters followed by a Roman numeral and ending in an Arabic number represent questions. Small case letters are answer values for the parent question. A specific answer or set of answers points to a specific follow up node, indicated by the direction arrows. Question nodes are of several types: yes/no; yes/no/uncertain; single selection multiple choice question; multiple choice questions; graphic representations of anatomy, e.g. to select affected joints in a patient with joint pain; and user entries, which can be entry of free text or entry from pull down menus. Nodes consisting of 3 letters represent sets of trees. Node PPU, for example, is a set of trees representing the pathophysiology of pulmonary disease as it applies to history-taking. The tree in Figure 1 will switch from acquiring a history concerning cardiac disease to focus on possible pulmonary disease when, for example, a patient's chest pain has the properties of pleuritic pain. The node PGL is a set of trees representing the pathophysiology of gastrointestinal disease, exclusive of liver disease, as it applies to history-taking. Nodes with 4 initial letters, a Roman numeral and ending in a capital letter represent single trees, which in turn represent single pathophysiologic issues. Node PGLHID represents a tree that questions the patient about swallowing disorders. Thus, when the history of chest pain (acquired by transit of the tree in Figure 1) indicates intermittent chest pain always precipitated by swallowing, the program will direct history-taking to the pathophysiology of swallowing disorders (node PGLHID) and then to a review of the gastrointestinal history (PGL). Nodes labeled complete end a string of questions; and depending on the arrangement of nodes at least one "Complete" node ends a tree.

As eluded to already, knowledge as trees for history-taking is broken-down by organ system and further as medical issues within an organ system. The elements acquiring the cardiac history, for example, are organized as separate trees for issues like hypertension, peripheral vascular disease, heart failure, coronary artery disease, and so on. Trees within an organ system and across organ systems are integrated as a single interview by a master tree so that patients are asked relevant questions about all systems no matter what their chief complaint, i.e., where in the set of trees an interview began. Moreover, no matter where the interview begins, e.g., independent of the organ system of the chief complaint or a switch in the pathophysiology pursued by history of the present illness, the program will be directed to acquire a history of all organ systems, a social history, a history of significant past medical events, a history of medications being taken, a history of known allergies and a family history. The master tree will direct the interview to a complete cardiac history as part of the review of systems for a patient with pleuritic pain or a patient with a swallowing disorder manifesting as chest pain. The program thus emulates history-taking by a physician, who may decide as the patient's story develops to switch a line of inquiry to pursue alternate pathophysiologies. This level of flexibility at run time is achieved through the mechanism of gating entry into every tree in the program.

The program "tests" whether a patient should be interviewed about every issue in the program as that issue is reached in the course of an interview. The "test" is based on answers to prior questions in the interview, which "gate" entry to every tree. Questions HUBHIB60 and HUBHIB33 gate the entry into the tree in Figure 1. One of these questions must be answered "Yes" (answer value a) for the tree to be entered. If the tree in Figure 1 is not entered, the interview proceeds to the next tree in the order of trees within the module NAP. The mechanism of gating enables the program to explore all aspects of the history that are significant for a given patient while avoiding redundancy and issues of no medical significance, as determined by answers to questions at each node in an interview.

Authors of content use the tree structures shown to develop their history-taking programs. The information in trees is compiled as machine-readable code, which directs the program at run time by calling questions from a database as the program instantiates one or another node. The program tested in this work comprised 8018 nodes representing approximately 12,000 data fields.

**Figure 2:**
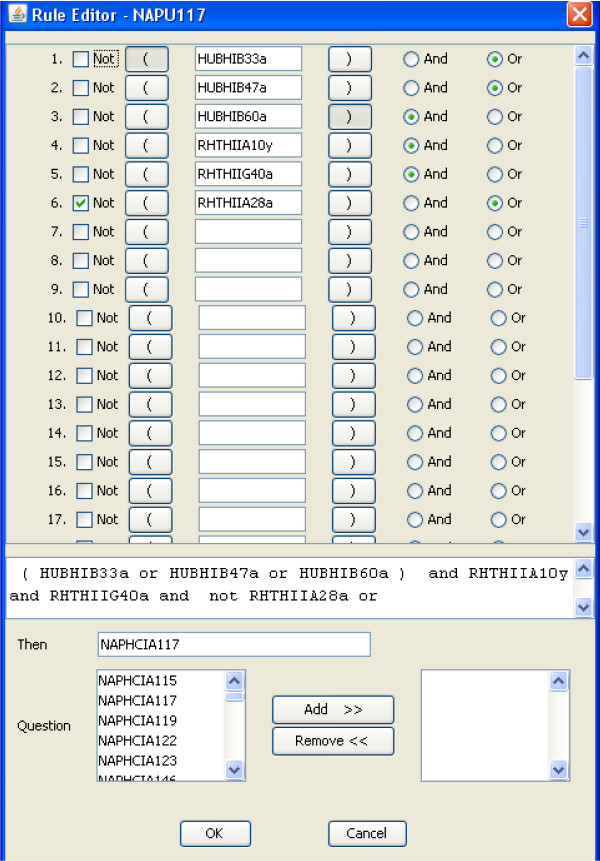
**Author interface for creating a rule (or inference) to interpret the significance of clinical data entered by patients**. This example uses the 3 operators available: and, or and not. The set of inferences runs automatically when an interview ends.

**Figure 3:**
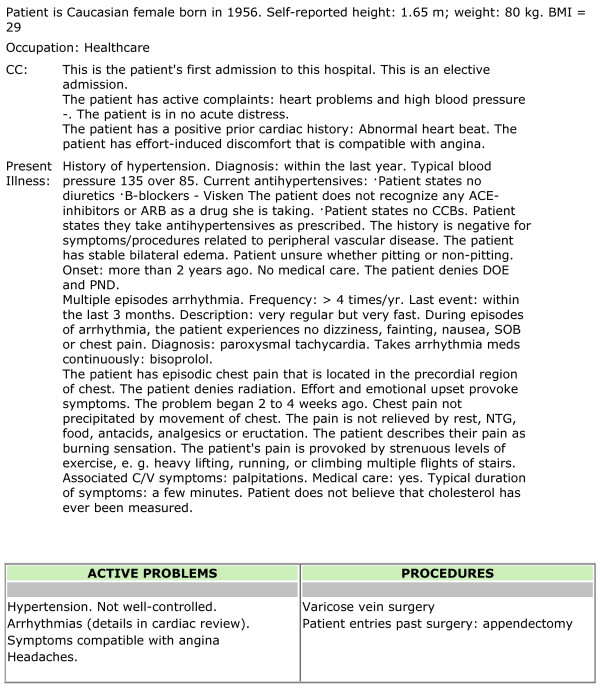
**Example of report compiled from interview data, as discussed in text**. C/V means cardiovascular; NTG means nitroglycerin.

At the end of an interview, the data acquired by the history-taking software are analyzed by a large set of inferences for which Figure 2 is an example. Rules use the Boolean operators AND, OR and NOT. The rules serve two functions. They organize the data as a clinical narrative; and they identify clinical problems. Figure 3 is an example of a report generated by the inferences within the program. Much of this report is narrative; but all key elements identified in an interview are reported as entries in Tables, e.g. Current medications, Table of Active Problems (Figure 3). Some of the problems identified in the table of Active Problems can be identified from entries into single data fields, as for example the presence of hypertension. The citation "Symptoms compatible with angina" is based, however, on a rule interpreting data according to pathophysiology. Note in Figure 2 that interpretation of clinical data to generate a possible diagnosis, e.g. "Symptoms compatible with angina" is concept-driven. "If statements" comprising a rule cite data fields not specific text.

### Deployment of software

Prior to deployment, the program was bench-tested extensively for errors in the logic of the trees, for errors in the flow of questions and gating of entry to trees and for errors in reporting of findings. In addition, the program was used by several hundred out-patients in the United States.

The software for acquiring the history operated from a central server behind a firewall within the IT department of the Robert-Bosch-Hospital (RBK, Stuttgart, Germany). Patients connected to the history-taking software via the hospital's Intranet. Patients selected their preferred language of German or English for the interview. After entering demographic data, they were asked to select a chief complaint from a set of menus. All subsequent questions were determined by the software as illustrated in Figure 1. Patients were able to review and change prior answers by moving the program backward one question at a time along the same branching pathways as the forward loop of questions. Moving backward erased answers to questions traversed in the backward direction. Just as the program determines when a specific tree ends (see figure 1), the master tree ends on a "Complete" node at the end of the family history. When the interview ends, all data are stored as codes in a structured, searchable database within the central computer. Data from computer interviews were extracted from compiled reports as in the example in Figure 3 not by review of individual data fields. Computer-acquired histories were available only to reviewers not to patients' physicians. Computer histories were extracted by D.Z.; physician histories were extracted from the hospital chart by N.B.

### Patient selection

Patients who were interviewed had been admitted emergently. Patient charts were not consulted prior to any interview; and the study nurse, who picked patients randomly for interview, had no knowledge of these patients before an interview and carried on no discussions with floor staff about a patient's clinical state. No demographic criteria were used to select patients except that interviews were limited to patients on general internal medicine, cardiology and nephrology services, which agreed to participate in the program. Patients were asked simply to participate in a test of a new program by which computers interview patients about their past and present health problems. No attempt was made to explain to patients the clinical value of the medical history. No patient who was asked to participate declined to be interviewed.

Ninety eight patients were interviewed by the computer program after a physician's history was recorded. Patients could choose between a computer workstation in the study unit of the clinic or use a laptop in bed, which had a wireless connection to the intranet of the hospital.

About half the patients had not used a computer previous to being interviewed. These patients were instructed in use of a computer mouse by the study nurse. All patients who agreed to computer interview were able to use the program. Only one patient, of those who started an interview, did not finish the interview, in this case because of severe pain secondary to gangrene of a limb. Hospital charts and records of computer interviews were reviewed in detail and reported here for 45 of these patients, who were interviewed by computer within 48 hours of a physician history. This cut-off was used because present illnesses precipitating hospital admission were not captured adequately by computer interviews for intervals between physician and computer interviews longer than 48 hours. The data relating to patient reaction to the computer interview and ease of use by patients includes all 98 patients, who filled out a questionnaire about their experiences with the computer-assisted interview. Responsible physicians did not know which patients were interviewed by computer and were not shown reports from these interviews.

### Data extraction and analysis

Data were extracted from hospital charts by N.B. and M.D.A.; data from the computer histories were extracted by D.Z., who tabulated comparisons between the 2 sets of records. Nurses at Robert Bosch Krankenhaus do not take medical histories in regard to allergies or adverse drug reactions. Pharmacists make no entries into charts and have no separate records of drug allergies or history of adverse drug reactions. Data on these issues either are obtained only by physician interview of the patient.

### Ethical approval

The local ethics committee and regulating government authorities approved the study (University of Tuebingen, Germany). Written informed consent was obtained from all patients. The study was registered (ClinicalTrials.gov: NCT00430755).

## Results

### Comparison of problems reported in computer but not physician histories

The data in Table 1 list, by organ system and component of the history, the medical problems reported in computer histories but not reported in physician histories for the 45 charts extracted. The computer histories reported 160 problems not recorded in physician histories (not counting allergies, possible adverse drug events and potential for adverse drug-drug interactions) or slightly more than 3.5 problems per patient across a range of organ systems involving acute and chronic issues and risks for further morbidity, as for example uncontrolled hypertension, unrecognized transient ischemic attacks (TIAs) and high risk of falling. We did not find a clustering of differences between computer and physician histories in a few patients but a wide distribution across all charts examined. Not unexpectedly, there was a larger numbers of discrepancies between computer-generated and physician histories in patients with a larger burden of disease.

**Table 1:** Significant problems reported by computer histories but not physician histories for 45 hospitalized patients

**Organ** **System/Issue**	**Problem Reported by Computer History but not Hospital Chart**	**Patients with Problem**
Heart	Symptoms heart failure (dyspnea on exertion, paroxysmal nocturnal dyspnea, marked fatigue, pitting edema) with no prior diagnosis	10
	Symptoms decompensated heart failure in patients with prior diagnosis	2
	"Cardiac asthma" versus reactive airway disease	4
	Prior diagnosis angina with chest pain	1
	Symptoms effort-induced angina versus reactive airway disease manifest as chest tightness	1
	Effort induced chest pain, no prior diagnosis angina	3
	Prior MI no invasive treatment	2
	Prior MI and angioplasty in last year	1
	Syncope	1
	Hypertension	8
	Uncontrolled hypertension by self-reported BP	3
	Arrhythmia	12
		
Peripheral	Symptoms compatible with intermittent claudication	5
Vascular	Symptoms intermittent claudication versus spinal stenosis	1
System	TIAs in patients with prior CVA	2
	TIAs in patient with no prior CVA	2
	History carotid artery surgery	1
	History peripheral arterial surgery	1
		
	Symptomatic asthma in patients with prior diagnosis but no rescue or control meds	5
	History treated TB	1
	Symptoms obstructive sleep apnea	1
	Symptoms sleep apnea	1
		
Pulmonary	34 old woman with daily cough and sputum throughout year. Onset as child. (Computer and physician history reported new onset fever and dyspnea with history pneumonia 4 months prior to admission)	1
		
GI	GERD, AM cough and hoarseness in patient with asthma	1
	Symptoms active ulcer disease in patient with prior upper GI bleed	1
	Daily aspirin use in patient with prior upper GI bleed	1
	Dysphagia in patient with scleroderma	1
	Erectile dysfunction	5
GU/GYN	Symptoms urinary obstruction in elderly men	7
	Stress incontinence (women)	2
	Symptoms estrogen deficiency in patients taking HRT	1
	Symptoms recurrent cystitis and back pain in woman	1
	Urinary incontinence since prostate surgery 1 year PTA plus 6 months dysuria, back pain and polyuria/polydypsia.	1
		
Endocrine/Metabolic	Uncertain thyroid status in patients treated previously for hyperthyroidism	3
	Polyuria/dyspsia, recent onset blurred vision	1
	Gout; no symptoms in last 1 year	1
	Polyphagia with no weight gain	1
	Diabetics with symptoms hypoglycemia	13
	Diabetics not monitoring or inadequately monitoring blood glucose	5
	Diabetics unaware of treatment regime for blood sugar	5
		
	Glaucoma	2
	Tunnel vision in patient with no prior diagnosis glaucoma	1
	Symptoms CNS vasculitis in patient with diagnosis SLE	1
	Episodic diplopia	1
	Tinnitus, hearing loss but no vertigo	1
	Current evidence major depression. No treatment.	2
	Major depression in setting of bereavement. No treatment.	1
	Current evidence moderate depression. No treatment	1
	Past history depression; not currently depressed; no meds	1
	Past history mania	1
	Prior diagnosis bipolar disorder not currently depressed	1
	Symptoms migraine; no prior diagnosis; positive FH migraine	1
Neurology	Multiple hospitalizations for falls; negative AUDIT score	3
	High risk for falling because of motor weakness, unsteady gait, past history falls not requiring hospitalization	10
	Episodic dizziness and abnormal gait in patient with positive AUDIT score	1
		
	Hodgkin's treated with XRT 3 years prior to admission. Now on chemo for 2^nd ^recurrence.	1
	Renal cell cancer treated with surgery and radiation 20 years prior to admission. No recurrence.	1
		
Oncology Rheumatology	Active rheumatoid arthritis on steroids	1
Life Style	Active smokers seeking help to stop smoking	2
	Positive AUDIT score	5
		
	Multiple 1^st ^and 2^nd ^degree relative with breast cancer	1
Family History	Multiple 1^st ^degree relatives with diabetes in patient with history gestational diabetes and recent onset chest pain provoked by emotional upset.	1
		
Vegetative	Night sweats	1
Symptoms	Weight loss > 10 pounds in non-dieting patient	1

The physician history was acquired prior to the computer history, which was obtained within the first 2 days of hospitalization.

### Comparison of problems reported in physician but not computer histories

The computer program asked patients to select a chief complaint from a series of menus, because the program does not have natural language processing or speech recognition. The sequence of items and menus of chief complaints appeared to impact what the patient selected. For example, several patients with a chief complaint of chest pain in physician histories selected more generic complaints from the computer menus, e.g., heart problems or breathing problems before seeing menus of complaints with chest pain, anginal equivalents, and so on. There thus was poor correspondence between the exact words of chief complaints recorded by physician and computer histories. On the other hand, there was good correspondence between the details of present illnesses collected by physician and computer histories across several chief complaints including patients reporting chest pain to their physician but heart or breathing problems in the computer interview. Exceptions to this are shown in Table 2, for which the chief complaint was chest pain in all but one patient. The key facts from physician and computer histories are illustrated here together with results of laboratory evaluations for each patient in whom the computer history failed in a significant way to mirror the physician history. The computer histories failed to report angina in 2 patients (lines 7 and 8) and failed to report acute urinary tract infection in another (line 9). One cannot say with certainty whether the physician history or computer history was a more accurate reflection of the patient's true state for patients in lines 1, 2, 3, 5 and 6; but the computer history is more compatible with the evaluations for these patients than the physician histories. Both sources of history failed to identify correctly the nature of the acute problem for the patient in line 4.

**Table 2:** Comparison of findings reported by physician histories and computer histories for 8 patients presenting to their physicians with a chief complaint of chest pain and for whom there were significant discrepancies between physician and computer histories of the present illness

**Age/sex**	**Physician-based Present Illness**	**Computer-based Present Illness**	**Diagnostic Outcome**
76/f	Angina	No acute disease	Negative cardiac cath
39/m	Exercise-induced angina	Progressive chest pain for 6 months radiating to L. shoulder, L. elbow and palpitations with emotional upset not effort. Patient also had effort-induced tightness of the chest and shortness of breath. ?Atypical angina and reactive airway disease	Negative cardiac cath; negative stress test.
50/f	Atypical angina	6 months SOB and tightness of chest with exercise and strong odors. No acute changes.	No diagnosis. No treatment.
49/m	Pleuritic chest pain; fatigue; 2 days fever and chills: pneumonia.	DOE progressive to dyspnea at rest at admission: heart failure	Pericarditis
54/m	Chest pain ? angina	No acute disease. Denied chest pain	No work up. Discharged in 1 day
85/f	New onset recurrent angina	DOE with daily chores. Old MI and denied recurrent chest pain.	Negative cardiac cath
77/m	Effort-induced chest pressure lasting 2 to 3 minutes and not relieved by NTG	No acute disease	Documented CAD by angiogram
76/m	2 years "angina" and dyspnea relieved by NTG	No acute disease	Documented CAD by angiogram
24/f	Acute UTI	No acute GU history	Pyelonephritis

It is not possible to examine the basis for discrepancies between computer histories and physician histories for the data in Table 1 because the physician leaves only a single record, which is the recorded history [see also Discussion]. The computer history leaves a searchable trail of what was asked, what was answered and the sequence of questions. The details of the computer histories for patients in line 7, 8 and 9 in Table 2 were analyzed to determine the basis for discrepancies between the reported computer histories and the recorded physician histories. The patient in line 7 entered in the computer history that he was admitted because of heart disease but answered "No" to a series of questions about chest pain and anginal equivalents provoked by effort or emotion or occurring at rest. The physician-acquired history for the patient mentioned in line 7 of Table 2 indicated a 2 year history of "angina" and dyspnea relieved by nitroglycerin. When interviewed by the computer-driven history program, the patient in line 8 selected they were admitted for heart disease and to have a heart catheterization, but denied a history of angina or heart attack. The patient answered "No" to the same series of questions cited for the patient in line 7 and "No" to questions about shortness of breath. The failure in interviewing the patient in line 9 was caused by a logic error. The software contained a set of questions appropriate to the patient's complaints suggesting pyelonephritis, as recorded by the physician history, but access to these questions in response to the patient's chief complaint of kidney problems was logically incorrect.

The problems in Table 3 were reported in physician histories but not computer histories. The failure of the computer histories to report the problems listed here reflect patient error in answering questions. The episodes of syncope reported in the medical charts were denied during the computerized history-taking as was the presence of diabetes. There is no coverage of skin lesions in the version of the program tested; so failure to report skin lesions is an inherent deficit. Failure to detect weight loss and night sweats in the instances cited reflects logic errors in the software, which asks about the systemic symptoms only in the context of specific disease entities not generically as part of a history of vegetative function. Both instances of weight loss and 1 instance of night sweats occurred in patients with end stage renal disease on dialysis, which was reported by the computer histories for both patients. The other instance of night sweats occurred in a patient with diabetes and decompensated alcoholic liver disease, which conditions were reported by the computer history for the patient.

**Table 3:** Problems reported in physician histories but not computer histories in 37 patients for whom data were extracted in Table 1 and for the 8 additional patients in Table 2

**Problem**	**Number of patients**
Night sweats	2
Weight loss (more than 5 pounds)	2
Single episode syncope	1
Purpura	1
Ulcerated skin lesions	1
Pruritis	1
Diabetes mellitus	2
Arrhythmia	1
Micturition syncope	1

### Drug allergies, adverse drug reactions and potential for adverse drug-drug interactions

The computer as compared with physician histories reported a greater incidence of allergies and possible adverse reactions to drugs [Table 4]. No documentation was available to assess the reason for discontinuing statins in 2 patients; CPKs were not available for the 2 patients with muscle soreness while taking statins. The data in Table 4 do not mean, of course, that the computerized history-taking detected all adverse events or drug reactions in the patients interviewed. The data show only the greater rate of detection of drug allergies and adverse reactions as compared with physician histories.

**Table 4:** Detection of allergies and possible adverse drug reactions in 45 in-patients for whom data are shown in Table 1

**Agent**	**Reaction**	**Physician History**	**Computer History**
Penicillin	Allergic with hives, giant hives and pruritis	2	4
Penicillin	Fever without skin reaction	0	1
Codeine	Allergic – not specified	1	0
Aspirin	Aspirin sensitive asthma	0	1
Latex	Allergic with giant hives, urticaria	0	1
Contrast Dye	Allergic with hives and itching	0	2
Peanuts/other nuts	Allergic with rash, hives, itching	0	1
Atorvastatin	Patients could not tolerate drug but denied muscle soreness or weakness or abnormal LFTs	0	2
Simvastatin	Muscle soreness	0	2
Simvastatin plus calcium channel blocker	Potential adverse interaction	0	1
Aspirin plus coumadin	Potential adverse interaction	0	2

### Patient satisfaction with the interview program

Patients reported a high level of satisfaction with the computer history; 69% believed their medical care would be enhanced after taking the computer-assisted interview [Table 5]. Almost half of these patients had not used a computer before. It was especially significant that only 3% of patients thought the interview was too long.

**Table 5:** Results of the questionnaire to get information about the experiences of patients after performing a computer-assisted interview

**Number of patients**	**N = 98**
***Age***	***58 ± 16 [18-89]***
***M – F***	***57/41***
	
Never used a computer before:	
- *men*	40%
- *women*	48%
Was it easy to understand the questions and answer them?	
- *Yes*	74%
- *No (for some questions)*	24%
- *No (for most of the questions)*	2%
If no, what questions were not easy to understand or to answer?	
- *Questions regarding medication*	74%
- *Questions regarding family history*	21%
- *Questions regarding cancer*	5%
What describes your experience with the Computer-assisted interview best:	
- *It is worthwhile to do since it is good for my further treatment*	69%
- *It is not helpful*	10%
- *Questions too personal*	3%
- *Takes too much time*	3%
- *Did not select an answer to this query*	15%

## Discussion

Bachman in 2003 reviewed the accumulated experience with computerized history-taking and found 61 instances of clinical tests of such programs [12]. Only 6 of these programs were in general medicine, and only 1 of these had a large database of medically relevant questions. Histories acquired by computer were compared with physician histories in 28 instances; and computer histories were determined to have provided more sensitive information than the physician histories in 25 of these 28 studies. These studies did not report details about the problems found by computer versus physician; and in some cases the findings were compilations of numbers of symptoms or numbers of questions asked by computerized history versus physician history. It is not possible to determine from these data the extent to which significant clinical information was not recorded by physician histories. Additionally, it is not extractable from the data to which extend a so called "checklist effect" solely is responsible for some of the found differences. This effect describes the phenomenon that a simple reminder of issues, which should be part of history taking, by a checklist can improve the quality of results.

A single study of computerized history-taking coupled to decision support found no advantages from the combination versus physicians providing usual care, measured by the number of problems detected in relatively young out-patients [13]. The report in reference [13] also lacked details for evaluating the disease burden in the study patients, the problems identified, whether the absence of differences in detecting problems by computer versus physician history applied to all patients and all problems and the relative contributions of the computerized history and decision support components to the measured end point of the study. Indeed, we believe the current report is the first to provide readers with clinical details of a history-taking program in general medicine with a database of questions in the tens of thousands and a patient-by-patient analysis of findings from computerized and physician history-taking and to document the nature of clinical information missing from physician histories. The software tested in the current study had not been refined previously by clinical testing with real patients, and in this sense was in an early stage of development. The data show, nevertheless, the significant extent to which physician histories as compared with computerized history-taking fail to acquire and/or record significant medical information on patients' medical status, that the deficiency in information gathering extends to acute and chronic issues and that the deficiency in collecting robust clinical information can be solved through computerized history-taking.

It is important to consider two issues related to the large false negative rate for physician history-taking. The first is whether this reflects false positive findings or unreasonable sensitivity to trivial symptoms by computerized history-taking. The data in the present report seem sufficient to exclude both possibilities. The computer-based findings are not the simple listing of symptoms or tabulation of questions asked. Problems were identified in the computer histories by automated analysis of answers to series of questions interpreted in the context of known pathophysiology. An obvious extension of the current work, however, is objective confirmation of findings by computerized history through direct, structured re-questioning of patients by an independent observer. The second issue is whether the computer histories benefitted from prior physician histories that triggered recall by patients of details about their health. There is no mechanism for excluding this possibility in acutely ill patients because medical ethics and liability law prevent prior interview of these patients by computer. The more important question is putting in place a method to maximize acquisition of clinically significant historical data. The present data show that a combination of a physician interview and a computer-based interview is a superior method for collecting information from patients as compared with physician interview alone whether or not the physician interview facilitates discovery of clinically significant problems by the computerized interview. We note that this combination can be achieved with little or no demand on doctors' time.

The accuracy of patient answers is a significant issue for computerized and physician history-taking. We mean by this mistaken entries not deliberate falsification of inputs. By testing computer programs against physician histories and objective measures of disease, points of common mistakes in computer entries can be identified. Three patients with diabetes answered "No" when asked directly, during the computer interview, whether they had diabetes. Two patients with objective evidence of coronary disease and physician histories compatible with effort-induced angina answered "No" when asked by computer about effort-induced chest pain and anginal equivalents. These false negatives could reflect failure by patients to read questions and answers carefully, poorly phrased questions and answers, mistaken selection of "No" when "Yes" was the intended answer, failure to comprehend prior diagnoses or intentional entry of false information. Editing of text and page design to facilitate usability and construction of feedback loops in the computer program will minimize chances for false negatives caused by misinterpretation of text, failure to read text carefully and data entry errors. Since the computer system can be configured to operate iteratively, the computer can re-interview patients on selected problems not detected during an initial interview but detected by objective measures of physiologic parameters. However, it also must taken into account that patients are not trained in pathophysiology as physicians are and therefore by building the program the programming doctors could not forecast the answers in precise manner. However, that impairment can be extinguished by repetitive testing of the program under real world conditions.

The demonstrable value of history-taking software as compared with physician-acquired histories depends on the training, knowledge and time available for history-taking by physicians as well as the burden of disease in a population. The differences between computerized and physician histories reported here might not be as large in tests in other facilities. On the other hand, the data indicate that most of the problems detected by computerized history-taking were chronic and had not been attended to prior to hospitalization. It seems clear that computerized history-taking will have value across an array of physicians.

## Conclusion

Even at this early stage of development, a combination of computerized and physician histories will be a valuable adjunct to practice because the combination uncovers more significant clinical problems than the physician history alone. In the acute situation, the computerized history can be obtain after the physician history and resuscitation of the patient, as the computer history will indicate a range of co-morbid states otherwise undetected. In the non-emergent setting, the computer history can be available to the physician at the time they see patients in order to focus on specific clinical issues.

## Competing interests

NB, MDA, and PF declare no competing interests. DZ is the holder of a U.S. patent for technology used in the software that was tested in this report and has a pending U. S. patent application for related technology. The issued patent is assigned and DZ is obligated to assign future patents to the IDM-foundation Institute for Digital Medicine, Stuttgart, Germany. These patents are assigned without rights to royalties.

## Authors' contributions

DZ was the designer of the software program, a significant contributor to the medical content of the software for history taking, extracted the data from computer records and compared these data with extracted clinical charts. MDA developed the study protocol, organized funding for the study and organized the legal frame. NB was responsible for review and data extraction from the hospital charts. MDA and PF were involved in design and conduct of the study. All authors contributed to writing of the manuscript.

## Source of funding

Funding for this work was provided by the Robert Bosch Krankenhaus and the Robert Bosch Stiftung. The funders had no role in planning or directing the clinical study and no influence on the data reported.

## Pre-publication history

The pre-publication history for this paper can be accessed here:


